# Genome-Wide Identification and Expression Analysis Unveil the Involvement of the Succinic Semialdehyde Dehydrogenase (*SSADH*) Gene Family in Banana Low Temperature Stress

**DOI:** 10.3390/ijms26073006

**Published:** 2025-03-26

**Authors:** Xiong Guo, Fengjie Yang, Xueying Zhang, Mengjie Tang, Kui Wan, Chunwang Lai, Zhongxiong Lai, Yuling Lin

**Affiliations:** Institute of Horticultural Biotechnology, Fujian Agriculture and Forestry University, Fuzhou 350002, China; wolikebear@163.com (X.G.); fengjieyang1001@163.com (F.Y.); zxyyingg@163.com (X.Z.); tangmengjie865@163.com (M.T.); 15565393028@163.com (K.W.); laichunwang@163.com (C.L.)

**Keywords:** banana (*Musa* app.), succinic semialdehyde dehydrogenase, low temperature stress

## Abstract

Banana (*Musa* spp.) is susceptible to low-temperature stress and other environmental stresses, which can hinder the growth and development. Succinic semialdehyde dehydrogenase (*SSADH*) is critical for GABA biosynthesis and plays a crucial role in plants. However, the *SSADH* genes of bananas have not been studied. This study found 19 *MaSSADHs*, 18 *MbSSADHs*, and 18 *MiSSADHs* from the banana genome. According to the phylogenetic tree, these genes can be categorized into five branches. This study cloned the *MaSSADH1-14* from banana. The subcellular localization assays of *MaSSADH1-14* in tobacco leaves confirmed that the presence of *SSADH* was not only localized mitochondrion but also localized chloroplast. The *cis*-elements of the *SSADH* gene family are related to the potential regulation of the banana *SSADH* gene family; their involvement in diverse stress responses. Transcriptomic data was utilized to examine the effect of *MaSSADH* genes under cold stress in bananas. The results of RT-qPCR were consistent with transcriptome data. These results showed that most *MaSSADHs* are passively responsive to low-temperature treatment. In addition, transient overexpression of *MaSSADH1-14* in *Nicotiana benthamiana* leaves resulted in the content of GABA increasing, indicating that *MaSSADH1-14* may be involved in the accumulation of GABA of banana. Collectively, these results improve knowledge of the *SSADH* gene family in banana and establish a basis for comprehending its biological roles in response to low temperatures.

## 1. Introduction

*SSADH* participates in multiple processes associated with plant growth, development, and stress response. *SSADH* (*ALDH5*) belongs to the plant acetaldehyde dehydrogenase (*ALDH*) superfamily in plants [1,2]. The *SSADH* gene family has been systematically analyzed in *A. thaliana*, *Sugarcane*, *Poplar*, *Setaria italica* L., *O. sativa*, and *V. vinifera*. Excess aldehydes will cause deleterious effects on organism metabolism. Therefore, the selective elimination of aldehydes is essential for cellular function [3]. The *ALDH* gene superfamily consists of enzymes that facilitate the NAD^+^ or NADP^+^-dependent transformation of several aldehydes into their respective carboxylic acids [4]. The *ALDH* gene family is able to dehydrogenate and oxidise aldehydes to carboxylic acids, thereby protecting plant cells from toxicity [5,6,7].

Low-temperature stress induces the accumulation of reactive oxygen species (ROS) in bananas, triggering membrane lipid peroxidation and compromising cell membrane integrity. In the process of active oxygen metabolism, excessive ROS causes damage to cells and even leads to tissue death [8]. Succinate semialdehyde dehydrogenase plays an important role in the GABA shunt. *SSADH* is an important enzyme that constitutes the GABA shunt [9]. The GABA shunt is a replenishment pathway to the tricarboxylic acid cycle (TCAC). It is crucial to the composition of the carbon skeleton and energy synthesis. It means that the GABA shunt has the function of balancing reactive oxygen species (ROS) and is indispensable in plant resistance to stresses [10]. *SSADH* has an established a role in preventing the accumulation of reactive oxygen intermediates and cell death [11] in *Arabidopsis thaliana*. Moreover, GABA shunt is the main anabolic pathway of GABA [10]. GABA can deal with various growth environments in plants [4]. Plants produce large amounts of GABA when exposed to extreme temperature stresses [12,13,14]. Increasing endogenous GABA levels in plants enhances resistance to low-temperature stress [15,16]. Therefore, we can enhance the cold resistance of plants through the GABA shunt pathway.

Banana (*Musa* spp.) is a significant commercial and food crop globally. The recurrent incidence of low-temperature stress in late winter and early spring has significantly impacted banana production and hindered the robust growth of the banana sector. Despite several investigations conducted by academics, the molecular mechanism governing the cold tolerance of bananas remains inadequately understood [17,18,19]. To date, there have been no prior publications regarding the systematic examination of the banana *SSADH* gene family. This study aims to comprehensively analyze the characteristics of the SSADH gene family in the banana genome and further investigate its response to low-temperature stress in bananas. We investigated the evolutionary relationships, conserved motifs, chromosomal positions, and promoter *cis-acting* elements of *SSADHs*. Additionally, several *MaSSADHs* were initially assessed for their role in modulating *Musa acuminata* tolerance to low-temperature stress. Furthermore, we verified subcellular localization of the key *SSADH* gene and investigated the effects of key *MaSSADH* genes transient expression endogenous GABA content changes in *Nicotiana Benthamian*. This study will aid research focused on the role of *SSADH* genes in plant growth and development, as well as in tolerance and resistance to low-temperature stresses, and will identify prospective candidate *SSADH* genes for breeding bananas that are tolerant to chilling temperatures.

## 2. Results

### 2.1. Identification and Physicochemical Property

This study removed duplicate incomplete sequences and sequences without corresponding structural domains from the sequences after multiple sequence comparison. Based on conserved domain integrity (Table 1), 19 *MaSSADHs*, 18 *MbSSADHs*, and 18 *MiSSADHs* were effectively discovered from the amino acid sequences of the *SSADH* gene family in *Musa acuminata*, *Musa balbisiana*, and *Musa itinerans*. They were named *MaSSADH1-1* to *MaSSADH1-19*, *MbSSADH1-1* to *MbSSADH1-18,* and *MiSSADH1-1* to *MaSSADH1-18* according to their gene ID and structure. The findings indicate that the lengths of *SSADH* genes range from 366 to 814 amino acids. The molecular weight (MW) ranged from 6.586 to 8.949 kDa. The predicted pI was between 5.21 and 9.36. An aliphatic index varied from 29.49 to 52.41. As for the physicochemical properties of the proteins, 42% of the *SSADH* gene family members GRAVY values are negative. In addition, most of the *SSADH* proteins did not have transmembrane domains, except *MaSSADH1-12* and *MbSSADH1-11*. The subcellular localization findings indicated that the majority of *SSADH* genes (94%) were situated in the mitochondrion and cytoplasm. But the *MaSSADH1-14*, *MbSSADH1-12*, *MiSSADH1-2,* and *MiSSADH1-11* were localized in the chloroplast.

### 2.2. Gene Structure, Conserved Motifs, Functional Domain Prediction, and Phylogenetic

**Figure 1:**
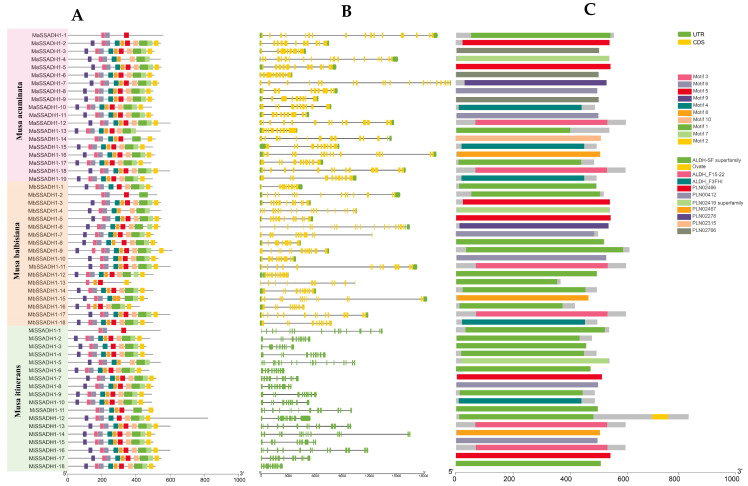
The picture describes gene motifs (**A**), gene structures (**B**), and protein domains (**C**) of banana *MaSSADH*, *MbSSADH,* and *MiSSADH* genes, respectively.

This study examined the structure of *SSADH* genes in banana for further investigation (Figure 1). We utilized the MEME to predict 10 conserved domain models for the *MaSSADH*, *MbSSADH*, and *MiSSADH* protein family members. The 10 conserved motifs of the banana *SSADH* proteins were analyzed using the MEME online website, and it was found that each *MaSSADH* contained motif 2. It is interesting that the *MaSSADH1-2* only has motif 2. Except for a few *MbSSADH* proteins with partial deletions, the rest contained motif 1, motif 3, motif 4, motif 5, and motif 7. Most of the *MiSSADH* proteins contained motif 1, motif 2, motif 3, motif 4, motif 5, motif6, motif 7, motif 8, and motif 10. These data reveal a significant level of sequence conservation within the *MaSSADH*, *MbSSADH*, and *MiSSADH* protein family. The examination of gene structure disclosed the length of the *SSADH* gene and the quantitative correlation between the coding sequence (CDS) and the untranslated region (UTR). *MaSSADHs* and most of *MbSSADHs* have UTR, except for *MbSSADH1-7* and *MbSSADH1-14*. The difference between the other two is that none of the members of the *MiSSADH* genome have UTR. The results demonstrate the variability of the banana SSADH protein sequences.

**Figure 2:**
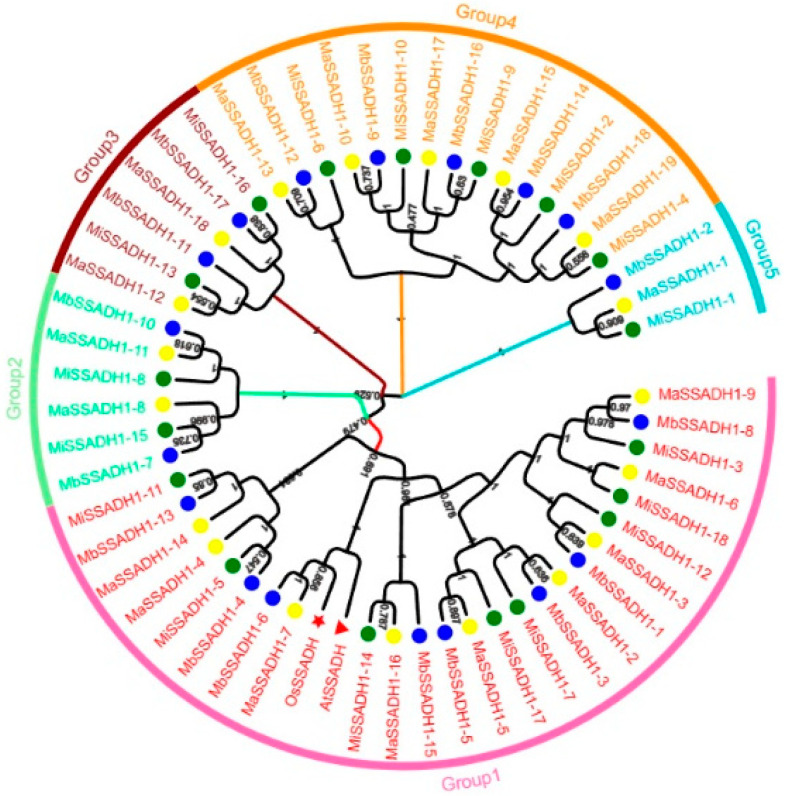
Phylogenetic tree for *Musa acuminata*, *Musa balbisiana*, *Musa itinerans*, *Arabidopsis thaliana,* and *Oryza sativa*. The SSADH proteins from *Musa acuminata*, *Musa balbisiana*, *Musa itinerans*, *Arabidopsis thaliana*, and *Oryza sativa* were firstly aligned using the ClustalW, and the phylogenetic tree was then constructed using MEGA X by the neighbor-joining method. A total of 1000 bootstrap replications were applied.

To investigate the phylogenetic relationship between the *SSADH* proteins and their homologs from other species, we created phylogenetic trees (Figure 2). The 57 sequences were divided into five groups, *MaSSADH1-9* to *MiSSADH1-11*, *MbSSADH1-7* to *MbSSADH1-10*, *MaSSADH1-12* to *MiSSADH1-16*, *MaSSADH1-13* to *MiSSADH1-4,* and *MbSSADH1-2* to *MiSSADH1-1*.

### 2.3. Chromosome Mapping and Collinearity Analysis

In order to comprehend the distribution characteristics and gene replication of the *SSADH* genes, the genomic annotation information of banana was employed to ascertain the chromosomal localization of the *MaSSADH*, *MbSSADH,* and *MiSSADH* genes.

**Figure 3:**
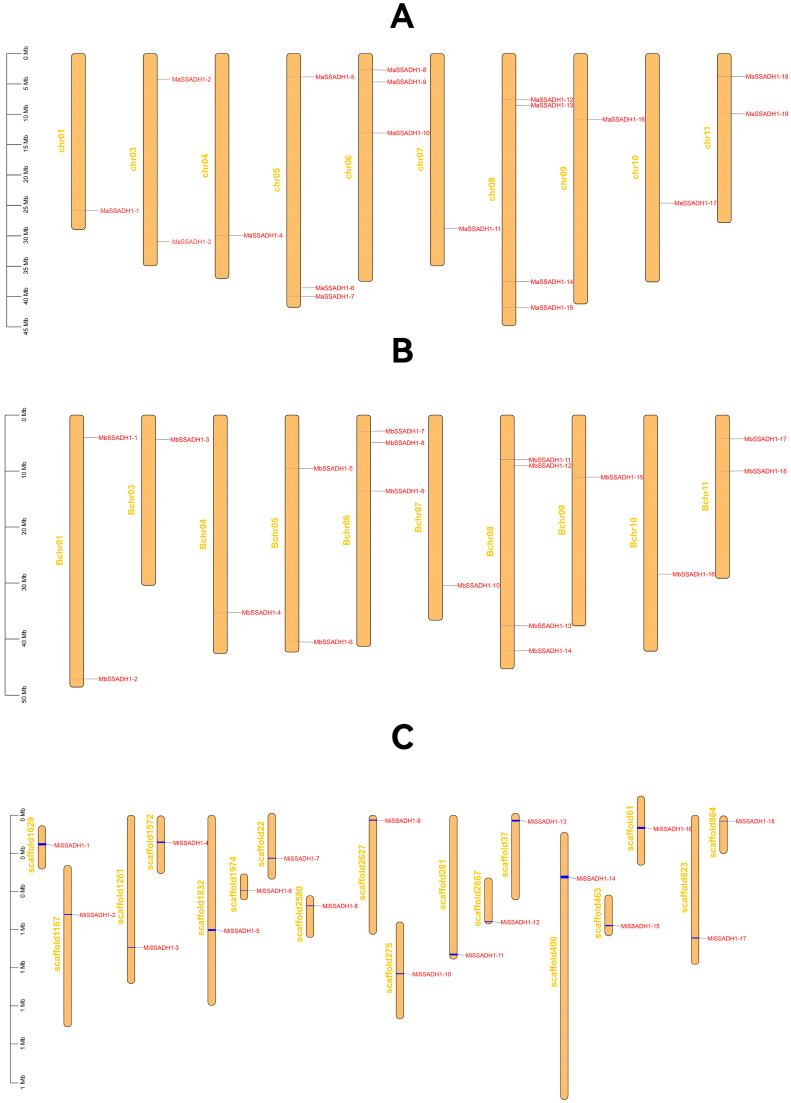
Chromosomal localization of *MaSSADH* (**A**), *MbSSADH* (**B**), and *MiSSADH* (**C**). Chromosomes are depicted in orange. Segmental duplication genes are linked by dark red lines.

**Figure 4:**
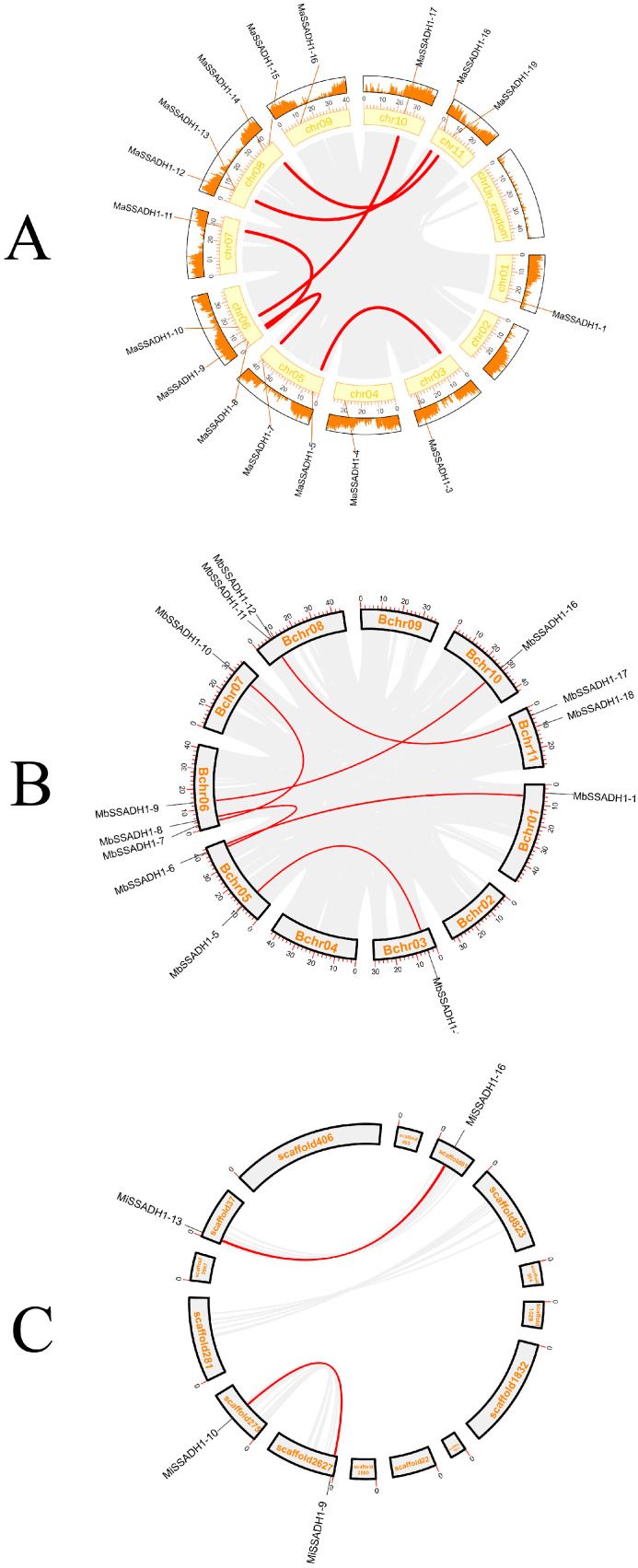
The collinearity analysis of *MaSSADH* (**A**), *MbSSADH* (**B**), and *MiSSADH* (**C**). Red lines: gene-duplication events of the *SSADH* gene family in banana. Grey lines:gene-duplication events of the other genes in banana.

We respectively mapped *MaSSADH*, *MbSSADH,* and *MiSSADH* to the *Musa acuminata*, *Musa balbisiana,* and *Musa itinerans* chromosomes to study their chromosomal distribution (Figure 3). As depicted in Figure 4, the 19 *MaSSADHs* exhibited an uneven distribution across 10 chromosomes, accounting for approximately 5/6 of the 12 chromosomes present in *Musa acuminata*. The 18 *MbSSADH* members were distributed on 10 chromosomes. Notably, the *M. itinerans* genome is a Scaffold-level genome. *MiSSADH* was localized on 18 scaffolds(S).

To understand gene-duplication events of the *SSADH* gene family collinear was used to analyze the whole genome (Figure 4). From the intragenome synteny analysis, there separately were six groups in *Musa acuminata*, six groups in *Musa balbisiana,* and two groups in *Musa itinerans* tandem.

**Figure 5:**
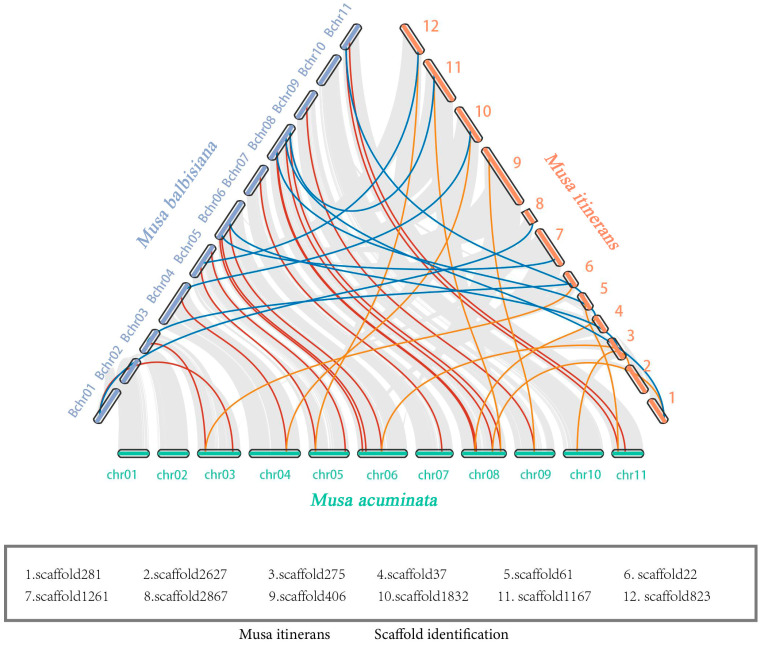
Collinearity visualization between *MaSSADHs*, *MbSSADHs,* and *MiSSADHs*.

To enhance comprehension of the evolutionary trajectory and source of the *SSADH* gene family, collinearity maps were generated comparing the three varieties of bananas (Figure 5). Based on the collinearity analysis among the three genomes of *Musa acuminata*, *Musa balbisiana,* and *Musa itinerans*, 26 gene pairs between *MaSSADHs* and *MbSSADHs*, 20 gene pairs between *MaSSADHs* and *MiSSADHs*, and 12 gene pairs between *MbSSADHs* and *MiSSADHs* were found. However, the sequence assembly of the *Musa itinerans* genome is fragmented and of weak continuity, being in the Scaffold(S) stage, the collinearity with the other two banana species is unclear. These results suggest that the *MiSSADHs* and *MbSSADHs* are closer to the *MaSSADHs* than between them.

### 2.4. Ka, Ks, and Ka/Ks of SSADH Gene Family

**Figure 6:**
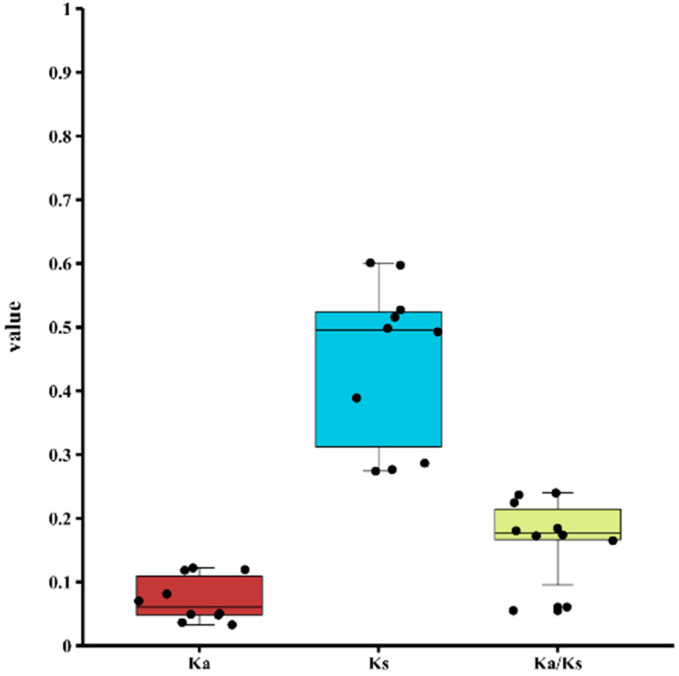
The Ka, Ks, and Ka/Ks value of the collinear *SSADH* gene pair.

The Ka/Ks ratio is often a significant signal of selection pressure in evolution. The study assessed the strength of natural selection by calculating the number of non-synonymous substitution sites (Ka) for *SSADH* homologous genes (Figure 6). The Ka/Ks ratios were determined, with the particular numbers presented in the Supplementary Table S2. Ka/Ks > 1 indicates a good selection effect. Conversely, Ka/Ks < 1 indicates the presence of a purifying selection effect. Neutral selection can solely be determined by a Ka/Ks ratio of 1. Consequently, 10 pairs of *SSADH* displayed low Ka/Ks values, with no homologous gene pair demonstrating a Ka/Ks ratio beyond 0.3. The Ka/Ks ratios ranged from 0.055 (*MaSSADH1-8*/*MaSSADH1-11*) to 0.236 (*MiSSADH1-9*/*MiSSADH1-10*), suggesting that the *SSADH* gene family has seen significant purifying selection following duplication.

### 2.5. Prediction Analysis of the SSADH Genes Promoter Cis-Acting Element

**Figure 7:**
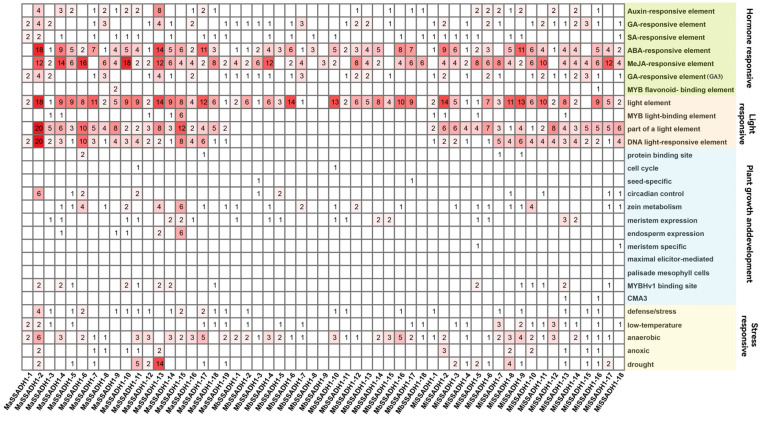
*Cis-acting* of *SSADH* promoter elements in *Musa acuminata*, *Musa balbisiana*, and *Musa itinerans*. The color depth of the square represents the number of cis-acting elements.

The cis-acting elements within promoters are generally related to abiotic stress responses, hormone responses, and developmental processes [7]. The detailed functional annotations of cis-acting elements are in the Supplementary Table S3. The *cis-acting* elements of *Musa acuminata* are involved in defense and stress responses, except for *MaSSADH1-1*, *MaSSADH1-7*, *MaSSADH1-8*, *MaSSADH1-13*, and *MaSSADH1-16*. But only *MbSSADH1-2*, *MbSSADH1-10,* and *MbSSADH1-11* have *cis-acting* elements involved in defense and stress responses in *Musa balbisiana*. And *MbSSADH1-1*, *MbSSADH1-7*, *MbSSADH1-9*, *MbSSADH1-13*, *MbSSADH1-15,* and *MbSSADH1-16* of *Musa itinerans* have this *cis-acting* element. In contrast to *Musa acuminata*, both *Musa balbisiana* and *Musa itinerans* possess components that participate in the response to gibberellin and methyl jasmonate. *MbSSADH1-5*, *MbSSADH1-8*, *MbSSADH1-11*, and *MbSSADH1-15* in *Musa balbisiana*, as well as *MiSSADH1-3*, *MiSSADH1-5*, *MiSSADH1-8*, *MiSSADH1-9*, and *MiSSADH1-17* in *Musa itinerans*, possess *cis-acting* elements associated with gibberellin response elements. These results suggest that the *SSADH* gene family possesses many biological functions and is crucial to light response, hormone regulation, and the growth and development in banana(Figure 7).

### 2.6. The Expression Analysis of MaSSADHs Under Low-Temperature Stress

**Figure 8:**
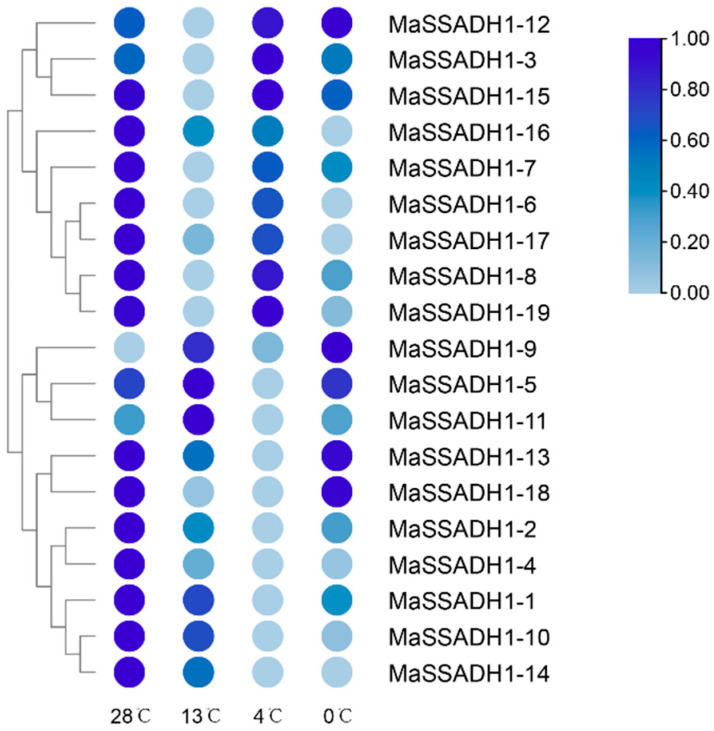
Expression analysis of banana *MaSSADH* family at different temperatures.

The expression levels of all 19 *MaSSADHs* were investigated thoroughly using a rigorous transcriptome analysis procedure based on public transcriptomic data of different temperatures of “*Sanmingyeshengjiao*”(Figure 8). The treatment at 4 °C led to the differential expression of 19 *MaSSADHs*. And two genes (*MaSSADH1-3* and *MaSSADH1-13*) were upregulated, and 17 were downregulated. The expression of the *MaSSADH1-1*, *MaSSADH1-2*, *MaSSADH1-4*, *MaSSADH1-10,* and *MaSSADH1-14* were differentially downregulated from 28 °C to 0 °C. The expression levels of *MaSSADH1-13* and *MaSSADH1-18* were downregulated from 28 °C to 4 °C and increased from 4 °C to 28 °C. And *MaSSADH1-5*, *MaSSADH1-9,* and *MaSSADH1-11* had higher expression in 13 °C.

**Figure 9:**
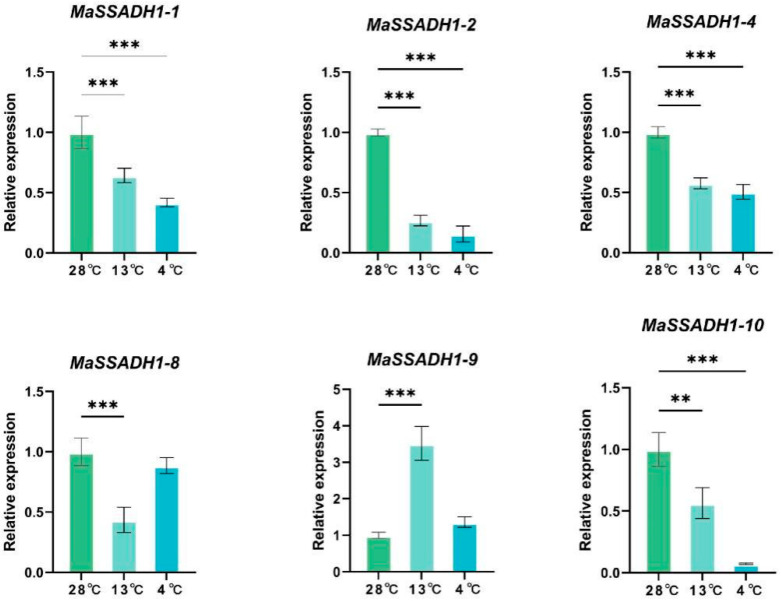

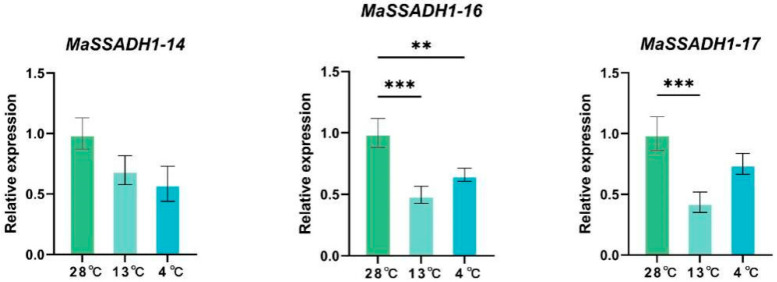
Expression patterns of nine *MaSSADHs* under temperature treatment at 28, 13, and 4 °C by qRT-PCR. The data represent the mean ± standard deviation (SD) of three replicates. ** and *** are significantly different from the control at *p* < 0.01 and 0.001, respectively.

To investigate the expression pattern of *SSADH* members under cold treatment, the expression patterns of nine randomly selected *MaSSADH* genes in different stages at low temperatures (4 °C, 13 °C, and 28 °C) were determined by qRT-PCR. The qRT-PCR results demonstrated that nine *MaSSADH* genes exhibited significant differences, showing expression patterns consistent with the transcriptome findings. And most of the genes were significantly downregulated under cold treatment (Figure 9). It is noteworthy that *MaSSADH1-9* were highly expressed under cold treatment. In addition, *MaSSADH1-1*, *MaSSADH1-2*, *MaSSADH1-4*, *MaSSADH1-9*, *MaSSADH1-10,* and *MaSSADH1-14* exhibited the highest expression levels at 13 °C. *MaSSADH1-8, MaSSADH1-16, and MaSSADH1-17* exhibited a comparatively elevated expression level following cold treatment at 4 °C.

### 2.7. Subcellular Localization and Transient Expression in N. benthamian Eaves of MaSSADH1-14

**Figure 10:**
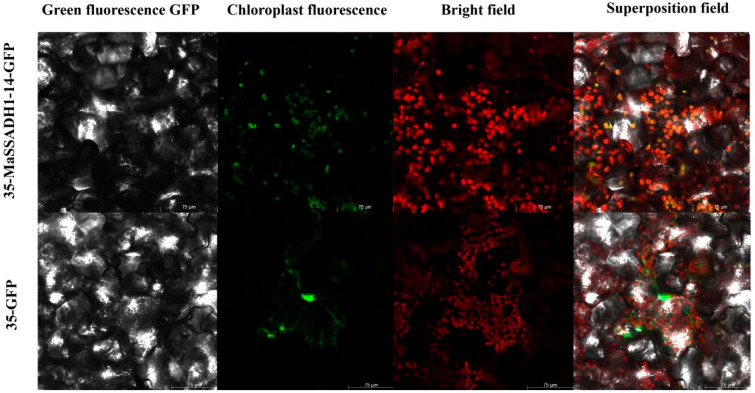
The subcellular localization of MSSADH1-14 protein. pCAMBIA1302- *MSSADH1-14*: GFP protein fusions transiently expressed in *Nicotiana benthamiana* leaf cells. Excitation light wavelength: GFP field: 488 nm, PM-rk field: 587 nm. Scale bar, 75 mm.

In previous studies, most of the *SSADH* were located in mitochondrion. However, some family members were predicted to target in other places. To explore the fact that the *SSADH* gene family are not only located in mitochondria, we randomly cloned *MaSSADH1-14*. One fusion vector was constructed and then transformed into tobacco leaf. And the GFP of the empty protein (35S: GFP) was used as a control group. The results show that *MaSSADH1-14* was detected as being localized to the chloroplast (Figure 10).

**Figure 11:**
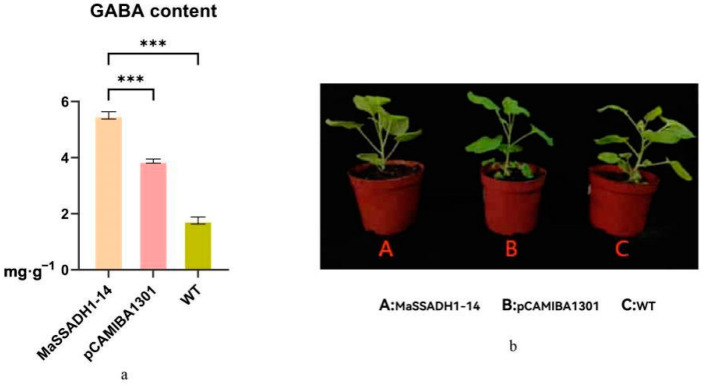
(**a**) Effect of infestation treatments on GABA content in *Nicotiana tabacum.* (**b**) Phenotypic map of lettuce under different treatments. *** are significantly different from the control at 0.001.

The information diversity of *SSADH* gene family members in banana are relatively limited. Based on the previous expression analysis, we selected *MaSSADH1-14*, hypothesizing that it may be involved in the accumulation of GABA in banana. Therefore, we used a transient expression system in *N. benthamiana* leaves to validate the functions of the genes. In this study, we selected *GUS* as a screening marker. Compared with the wild type, the GABA composition was changed greatly in the transgenic tobacco (from 1.76 mg/g to 5.52 mg/g). The content of GABA was remarkably higher in positive transgenic tobacco than in wild-type (*p* < 0.001), indicating that the *MaSSADH1-14* may contribute to the accumulation of storage GABA in *N. benthamiana* leaves (Figure 11).

## 3. Discussion

Banana often suffers from external low-temperature stresses in winter [20]. It causes huge economic losses to the banana industry. Cell membrane damage was considered the initial reaction of chilling injury. Low-temperature stress induces the outbreak of reactive oxygen species in banana, accompanied by a decrease in antioxidant capacity. These can lead to impairment of banana cell membrane function [21]. GABA shunt balances reactive oxygen species (ROS). With the GABA shunt, it is possible to generate GABA. GABA significantly inhibits the low-temperature injury and enhances the anti-oxidative enzyme activities, such as superoxide dismutase, catalase, ascorbate peroxidase, and dehydro-ascorbate reductase [22]. *SSADH* is a critical enzyme in the GABA shunt pathway; it is a class of dehydrogenases functioning primarily in the mitochondria [9]. For example, mutation of this gene has been shown to cause death in *Arabidopsis thaliana* [23], where it produces the metabolite γ-hydroxybutyrate (GHB), which is currently thought to be associated with abiotic stresses in plants [24]. Furthermore, *SSADH* is a substantial subfamily of the *ALDHs* family. In plants, *ALDHs* are involved in abiotic stress tolerance [25]. This paper is the first public investigation and identification of *SSADH* through banana genomic data, enhancing the comprehension of the *SSADH* gene family function under cold stress in banana.

The majority of currently cultivated bananas originated from inter- or intraspecific crosses between two wild diploid species, *Musa acuminata* and *Musa balbisiana*, and their subspecies [26]. *Musa balbisiana* is commonly used in breeding studies because of its resistance characteristics to cold, while *Musa itinerans* has been shown to be one of the most resistant species in the genus Plantago and can be used as material for studies on banana’s resistance to cold [27]. In this study,19 *MaSSADHs*, 18 *MbSSADHs,* and 18 *MiSSADHs* were identified from the three banana genomes. Nonetheless, substantial disparities exist in the quantity of *SSADH* gene family members among species, likely due to gene duplication or deletion events during evolutionary history. There are 1, 2, 1, 1, 1, and three *SSADH* genes in *A. thaliana* [7], *Sugarcane* [28], *Poplar* [29], *Setaria italica* L. [29], *O. sativa* [30], and *V. vinifera* [30]. It has been noted that three whole-genome duplication (WGD) events transpired in the banana genome over its evolutionary history [31]. Gene polyploidy is widespread and recurrent in plant evolution, and polyploidy produces a large number of duplicated genes, which are adaptations to the external environment and evolve genetically innovative material with higher levels of function [30]. Therefore, the higher number of members of the *SSADH* gene family in banana may be a response mechanism that occurred in banana during the evolutionary process of adapting to the environmental changes.

And 58% of the *SSADH* gene family members are hydrophilic proteins, which are favourable for plant adaptation to abiotic stresses [31], and it is hypothesised that the proteins play an important role in stressful environments. In the prediction of subcellular localisation of the proteins, most of the members of the banana *SSADH* gene family were localised in mitochondria, which is the same as in *Arabidopsis thaliana*, rice, and other plants, and is presumed to be associated with mitochondrial metabolic functions [7]. Furthermore, *MaSSADH1-14*, *MbSSADH1-12*, *MiSSADH1-2,* and *MiSSADH1-11* are localised in the chloroplast and is speculated to be involved in chloroplast formation, thereby affecting photosynthesis. It is proven that *MaSSADH1-14* is in the chloroplast through subcellular localization.

The structure of exons and introns affects the function of proteins and the expression and regulation of genes, which means that exons and introns enable genes to play an important role in organisms and thus in the maintenance of life [32,33]. Our findings indicate that all *SSADHs* possess introns, suggesting that this characteristic may have been evolutionarily conserved and potentially hold functional significance. These genes may utilize introns to generate unique splice variants, hence modulating gene function for specific developmental stages and tissue types [34]. Furthermore, members of the *SSADH* gene family in *Musa acuminata* and *Musa balbisiana* have UTR, except for *MbSSADH1-13*. However, all *SSADH* gene family members in *Musa itinerans* do not have a UTR, indicating that the structure of the *SSADH* gene has diversified between different genomes of banana. Most members of the conserved motifs in these three gene families in banana have the same motif, indicating that there is a high degree of consistency in the structure of the members of this gene family and that the members are highly conserved.

A phylogenetic tree analysis of *SSADH* family members from *Arabidopsis* and rice revealed their classification into five subfamilies. Interestingly, over half of these members were grouped together with those from banana, indicating a close relationship among the family members across these species. However, evolutionary selection processes have led to distinct classifications. To assess the evolutionary trajectory of the *SSADH* gene family, the Ka/Ks values of 10 pairs of duplicated tandem genes were calculated. Remarkably, all sequences exhibited Ka/Ks values below 1, suggesting a prevalence of negative selection or purifying selective pressure within the *SSADH* gene family. This observation underscores the high conservation of the *SSADH* gene family throughout evolution, implying a consistent functional role across species.

Gene duplication is a crucial driving force in the evolution of genomes and the genetics of species [35]. The primary factors contributing to gene family amplification in plants are segmentation and tandem duplication [36]. Gene duplication can not only add new members to the gene family but also enrich the function of the gene family, which greatly promotes the genetic evolution of various organisms [37]. Tandem and segmental replication events were identified in the *SSADH* gene family, suggesting that gene replication was a significant factor in the expansion of *SSADH* gene family members in banana. The results of the syntenic analysis of *SSADHs* among *Musa acuminata*, *Musa balbisiana,* and *Musa itinerans* show that there were three collinear gene pairs in *Musa acuminata* and *Musa balbisiana*, *Musa acuminata,* and *Musa itinerans,* and five collinear gene pairs in *Musa balbisiana* and *Musa itinerans*.

The many types of *cis-acting* elements in the gene promoter suggest that the gene may exhibit diverse roles in response to stress [38]. The promoter prediction results show that the cis-regulatory element of the three bananas had many response elements linked to photo responses, plant hormone responses, and environmental stress. This shows that it plays a significant role in the growth and development of plants [39,40]. Plant hormones are essential signaling molecules that regulate plant development, growth, and defense systems [41,42,43]. Recent research indicates that the exogenous administration of plant hormones such as abscisic acid, brassinosteroids, gibberellins, auxins, cytokinins, jasmonic acid, and ethylene can markedly improve cold tolerance [44]. And three banana genomes contain elements related to low-temperature response or resistance to adversity, suggesting that this clade plays an important role in resistance to adversity and stress response. Additionally, the promoter region of the *SSADH* gene has a significant quantity of light-responsive components, suggesting a possible association with circadian control.

Gene expression is closely related to gene function [45]. The expression was significant alter under low temperature of *MaSSADH* genes, except *MaSSADH1-5*, *MaSSADH1-6,* and *MaSSADH1-12*. This indicates that the *SSADH* gene family is regulated by temperature in banana. As the temperature decreased, the expression of these 16 members were downregulated, except for *MaSSADH1-10* and *MaSSADH1-14*. The work indicates the substantial regulatory function of the *SSADH* gene family in banana responses to low-temperature stress.

RNA-seq is typically employed to investigate gene function and structure comprehensively, elucidating the molecular mechanisms behind specific biological processes and disease manifestation [46,47]. The relative gene expression of some *SSADH* gene family members in banana was examined by qRT-PCR under low-temperature treatment. Our analysis indicated that of the nine *MasSSADHs* examined, the majority of genes were downregulated in response to cold stress, but only *MasSSADH1-9* was upregulated. The research suggested that these *MaSSADHs* are probably in response to low-temperature stress. It means that *SSADH* genes have potential applications in cold-resistant breeding. However, its specific mechanism of action still needs to be further explored.

## 4. Materials and Methods

### 4.1. Plant Material and Low Temperature Treatment

The five- to seven-leaf banana used in this study was from the Institute of Horticultural Biotechnology, Fujian province, China. These bananas were divided into three groups. The banana was positioned in a thermostatic incubator (Jiangnan Instrument Factoy, Ningbo, China) at 4 °C, 13 °C, and 28 °C. We collected the leaves of samples at 24 h.

### 4.2. Identification, Physicochemical and Phylogenetic Tree

The genome information of *SSADH* genes were acquired from Banana-Genome-Hub (https://banana-genome-hub.southgreen.fr/, accessed on 15 April 2023) [48]. The *AtSSADH* sequences were from TAIR (https://www.arabidopsis.org/, accessed on 5 July 2023). To obtain the potential *SSADHs* in banana, the *SSADH* gene family member of *Arabidopsis thaliana* was used as query sequences to obtain preliminary candidate genes using BLAST (2.12.0) in banana (E-value ≤ 1 × 20^−5^). Next, the hidden horse model PF00171 [49] was used to search *SSADH genes.* And tbtools [50] was used to search the genome database. We combined these results to CD search (https://www.ncbi.nlm.nih.gov/Structure/cdd/wrpsb.cgi, accessed on 27 July 2023). It was confirming the candidate *SSADH* [51]. ExPAsy (https://web.expasy.org/protparam/, accessed on 28 April 2023) as a tool to predict physicochemical of *SSADH* proteins [52]. Plant-mPLoc (http://www.csbio.sjtu.edu.cn/bioinf/plant-multi/, accessed on 22 March 2023) was used for the subcellular locations and [53]. TMHMM Server (https://services.healthtech.dtu.dk/service.php?tmhmm-2.0, accessed on 23 March 2023) was used for the transmembrane analysis [54]. The study used MEGAX to examine *SSADHs* from *Musa acuminata*, *Musa balbisiana*, *Musa itinerans*, rice, and *Arabidopsis* to determine their phylogenetic relationship. MEGAX [55] was utilized to build a phylogenetic tree.

### 4.3. Gene Structure, Motifs and Domains

The exon-intron architecture was inferred by delineating the gene structure of all potential banana *SSADH* genes utilizing TBtools (2.056). The conserved motifs of the *SSADH* family in banana species were examined using the MEME online tool (https://meme-suite.org/meme/doc/meme.html, accessed on 20 March 2025), with the motif parameter configured to 10 [56]. The TBtools mapping program helped one to see the gene structure. TBtools displayed the chromosomal sites of banana in line with the genomic annotation file.

### 4.4. Cis-Acting Element Prediction

We submitted the sequences of *SSADH* genes to PlantCART (http://bioinformatics.psb.ugent.be/webtools/plantcare/html/, accessed on 3 May 2023). The 2000 bp upstream sequences were employed to forecast the *cis-acting* elements. Subsequently, tbtools serves as a tool to illustrate the results of PlantCART [57].

### 4.5. Chromosome Mapping and Collinearity

Drawing distribution map and length scale of chromosome by TBtools software [50]. We respectively analyzed *SSADH* gene family collinearity and gene replication by TBtools (v2.056) in three bananas. The relationships were verified and visualized by the JCVI [58] for collinearity analysis in three bananas.

### 4.6. Ka/Ks Values Analysis

The Ka/Ks values is a genetic metric that evaluates the impact of selection pressure on these genes. The study utilized TBtools (v2.056) software to calculate it.

### 4.7. Transcriptome Data and qRT-PCR

To investigate the expression profiles of *MaSSADHs* across various temperature, the transcriptome data of *MaSSADH* genes were retrieved from the bioproject database [59]. The raw data was deposited in NCBI-SRA database (SRA: SRS3320042). And the expression heatmap was drawn using tbtools to analyse the expression of the *MaSSADHs*.

Differentially expressed *SSADH* family genes were verified using qRT-PCR. The primers were used IDT to design (https://sg.idtdna.com/pages, accessed on 20 March 2025) [60]. The nine *SSADH* genes were selected randomly. *CAC* was used as a reference gene [61]. The relative expression levels of *MaSSADHs* were calculated using the 2^−∆∆Ct^. Prism GraphPad 9.5 was employed to assess the significance test among samples. All data were analyzed using GraphPad Prism 9.5 for one-way ANOVA and Dunnett’s multiple comparison test (* *p*  <  0.05, ** *p*  <  0.01, and *** *p*  <  0.001).

### 4.8. Subcellular Localization

We used snapgene 7.1 to design primers of pCAMBIA1302-*MaSSADH1-14:GFP* (Table S1). The *MaSSADH1-14* was cloned without stop codon into pCAMBIA1302 vector. *SpeI* and *NcoI* were utilized to linearize pCAMBIA1301. Subsequently, we introduced the 35S: *MaSSADH*-GFP construct into tobacco leaves. The vector devoid of the gene were utilized as controls. The overexpression vector pCAMBIA1302-*MaSSADH1-14*: GFP was successfully transformed into tobacco epidermal cells, while pCAMBIA1302: GFP alone was transformed to use as a control. We cultivated *N. benthamiana* in the dark for 24 h after injection into the abaxial epidermis [62]. The Olympus-FV1200 (Japan) was employed to examine the GFP fluorescence signals post-treatment.

### 4.9. Transient Expression of in N. benthamiana Leaves

The snapgene 7.1 was the tool to design primers of pCAMBIA1301-*MaSSADH1-14* (Table S1). *XbaI* and *SalI* were utilized to linearized pCAMBIA1301. The three experimental treatment groups were blank group (no injection), overexpression group, injection group (injection of pCAMBIA1301-*MaSSADH1-14*) and empty vector (injection with pCAMBIA1301). Each treatment had 30 *Nicotiana benthamiana*. We extracted DNA to verify the positive transformation. Samples were collected to measure the GABA content after 48 h of treatment.

### 4.10. Measurement of the Contents of GABA

We used the GABA assay kit (Herui, Fuzhou, China) to measure the content of GABA. The assay was measured at an absorbance at 640 nm, which is based on the reaction of phenol and sodium hypochlorite with GABA.

## 5. Conclusions

The study found and analyzed 19 *MaSSADH*, 18 *MbSSADH*, and 18 *MiSSADH* genes using banana genomic data. Most of the *SSADH* genes are in response to low-temperature stress. The transient expression of *MaSSADH1-14* underscores its importance in regulating GABA accumulation. These results indicated the important role of the *SSADH* gene family in low-temperature responses. This will serve as a foundation for subsequent research on the role of *SSADH* genes in bananas under low-temperature conditions. Furthermore, this study provides a theoretical basis and molecular mechanism support for breeding cold-tolerant banana varieties.

## Figures and Tables

**Table 1:** Physicochemical properties of SSADH proteins.

Gene Name	Gene ID	Size/Amino Acid	MW	pI	Instability Index	Aliphatic Index	GRAVY	Signal Peptide	THM	Subcellular Location
*MaSSADH1-1*	Ma01_g22760	553	61,356.54	6.5	37.2	93.45	−0.11	NO	0	Mitochondrion
*MaSSADH1-2*	Ma03_g06120	539	58,811.42	7.15	36.22	90.54	−0.06	NO	0	Mitochondrion
*MaSSADH1-3*	Ma03_g27650	502	54,493.45	5.79	37.69	85.52	−0.051	NO	0	Cytoplasm
*MaSSADH1-4*	Ma04_g28920	538	57,767.19	7.58	40.36	85.99	−0.057	NO	0	Mitochondrion
*MaSSADH1-5*	Ma05_g05010	542	58,806.33	6.71	31.05	90.2	−0.054	NO	0	Mitochondrion
*MaSSADH1-6*	Ma05_g27170	501	54,588.82	5.89	37.46	88.04	−0.008	NO	0	Mitochondrion
*MaSSADH1-7*	Ma05_g29200	530	56,816.59	7.45	33.85	92.98	0.056	NO	0	Nucleus
*MaSSADH1-8*	Ma06_g03680	496	53,168.62	7.11	36.86	93.61	0.039	NO	0	Mitochondrion
*MaSSADH1-9*	Ma06_g06420	501	54,700.81	5.99	31.66	84.13	−0.053	NO	0	Cytoplasm
*MaSSADH1-10*	Ma06_g19080	487	53,283.31	9.36	34.71	101.72	0.068	NO	0	Cytoplasm
*MaSSADH1-11*	Ma07_g20820	497	53,298.52	6.39	38.33	90.62	−0.008	NO	0	Cytoplasm
*MaSSADH1-12*	Ma08_g10390	595	65,813.21	6.9	30.76	94.67	0.083	YES	1	vacuole
*MaSSADH1-13*	Ma08_g11500	537	59,878.98	8.98	52.41	108.23	0.077	NO	0	Cytoplasm
*MaSSADH1-14*	Ma08_g24330	509	54,433.77	5.63	35.36	95.74	0.113	NO	0	Chloroplast
*MaSSADH1-15*	Ma08_g30610	493	53,446.84	7.63	38.35	95.01	0.022	NO	0	Cytoplasm
*MaSSADH1-16*	Ma09_g15520	505	55,101.55	5.21	34.09	92.75	−0.018	NO	0	Peroxisome
*MaSSADH1-17*	Ma10_g10980	484	52,750.29	8.95	32.72	100.52	0.069	NO	1	Cytoplasm
*MaSSADH1-18*	Ma11_g04820	594	65,526.86	6.94	32.3	93.72	0.099	NO	1	vacuole
*MaSSADH1-19*	Ma11_g10470	493	53,481.1	8.65	37.08	96.39	0.013	NO	0	ER
*MbSSADH1-1*	Mba01_g05390	491	53,233.89	5.6	36.97	84.26	−0.073	NO	0	cytoskeleton
*MbSSADH1-2*	Mba01_g32920	516	57,139.74	6.93	36.82	92.98	−0.131	NO	0	Mitochondrion
*MbSSADH1-3*	Mba03_g06130	539	58,851.52	6.81	36.54	91.82	−0.045	NO	0	Mitochondrion
*MbSSADH1-4*	Mba04_g29540	538	57,592.98	6.77	40.62	86.17	−0.039	NO	0	Mitochondrion
*MbSSADH1-5*	Mba05_g12480	542	58,790.34	6.71	30.24	90.55	−0.045	NO	0	Mitochondrion
*MbSSADH1-6*	Mba05_g28500	535	57,348.96	5.64	31.95	93.01	0.063	NO	1	Nucleus
*MbSSADH1-7*	Mba06_g03350	498	53,471.08	7.85	35.27	93.23	0.029	NO	0	cytoskeleton
*MbSSADH1-8*	Mba06_g06030	519	57,033.67	5.69	34.15	89.65	0.02	NO	0	cytoskeleton
*MbSSADH1-9*	Mba06_g18010	607	66,434.55	8.79	34.3	99.13	0.12	NO	0	plas
*MbSSADH1-10*	Mba07_g19440	525	56,537.46	6.16	38.31	93.96	0.09	NO	1	Cytoplasm
*MbSSADH1-11*	Mba08_g09970	595	65,859.14	6.22	29.49	95.16	0.089	YES	1	vacuole
*MbSSADH1-12*	Mba08_g10980	494	55,125.19	8.87	45.12	101.23	0.064	NO	0	Chloroplast
*MbSSADH1-13*	Mba08_g23970	366	39,846.99	5.94	38.95	99.62	0.056	NO	1	Cytoplasm
*MbSSADH1-14*	Mba08_g30180	493	53,645.11	7.16	37.82	93.81	0.012	NO	0	Cytoplasm
*MbSSADH1-15*	Mba09_g14730	463	50,612.74	5.52	35.67	94.21	0.03	NO	0	Peroxisome
*MbSSADH1-16*	Mba10_g09530	415	45,476.94	9.24	33.29	97.73	0.012	NO	1	Cytoplasm
*MbSSADH1-17*	Mba11_g04730	594	65,549.94	6.94	32.84	93.72	0.094	NO	1	vacuole
*MbSSADH1-18*	Mba11_g09820	493	53,542.06	8.06	37.14	95.42	−0.005	NO	0	ER
*MiSSADH1-1*	Mi_g000749	537	59,571.34	6.08	37.03	93.5	−0.117	NO	0	Cytoplasm
*MiSSADH1-2*	Mi_g002257	476	52,002.4	8.07	40.14	97.77	0.049	NO	0	Chloroplast
*MiSSADH1-3*	Mi_g003077	456	49,936.31	5.88	31.2	85.18	−0.081	NO	0	Cytoplasm
*MiSSADH1-4*	Mi_g006293	493	53,582.16	8.51	36.48	96	−0.01	NO	0	ER
*MiSSADH1-5*	Mi_g008747	538	57,800.3	7.56	40.86	87.06	−0.056	NO	0	Mitochondrion
*MiSSADH1-6*	Mi_g009992	472	52,510.92	8.14	48.8	99.17	0.034	NO	0	Cytoplasm
*MiSSADH1-7*	Mi_g011667	511	55,684.91	6.2	32.23	92.43	−0.038	NO	0	Cytoplasm
*MiSSADH1-8*	Mi_g014343	497	53,341.56	6.11	39.1	91.23	0.006	NO	0	Cytoplasm
*MiSSADH1-9*	Mi_g014566	487	53,083.58	8.6	33.37	100.49	0.054	NO	1	Cytoplasm
*MiSSADH1-10*	Mi_g015510	487	53,289.34	9.33	33.25	101.72	0.073	NO	0	Cytoplasm
*MiSSADH1-11*	Mi_g016157	498	53,257.46	5.9	34.52	95.3	0.12	NO	0	Chloroplast
*MiSSADH1-12*	Mi_g016499	814	89,491.01	5.83	48.11	73.26	−0.331	NO	0	Nucleus
*MiSSADH1-13*	Mi_g019946	595	65,699.07	6.92	29.76	95.5	0.092	NO	1	vacuole
*MiSSADH1-14*	Mi_g021384	505	55,125.65	5.27	34.25	91.6	−0.022	NO	0	Peroxisome
*MiSSADH1-15*	Mi_g023084	496	53,167.63	7.11	36.92	93.41	0.037	NO	0	cytoskeleton
*MiSSADH1-16*	Mi_g026726	594	65,595.99	6.94	33.39	93.06	0.09	NO	1	vacuole
*MiSSADH1-17*	Mi_g030503	542	58,743.3	6.9	30.88	90.39	−0.038	NO	0	Mitochondrion
*MiSSADH1-18*	Mi_g030913	509	55,443.88	5.78	36.45	88.76	0.02	NO	0	Cytoplasm

## Data Availability

The data sets presented in this study can be found in online repositories. The names of the repository/repositories and accession number(s) can be found in the article/Supplementary Materials.

## Supplementary Materials

The following supporting information can be downloaded at: https://www.mdpi.com/article/10.3390/ijms26073006/s1.
